# KLF17 empowers TGF-*β*/Smad signaling by targeting Smad3-dependent pathway to suppress tumor growth and metastasis during cancer progression

**DOI:** 10.1038/cddis.2015.48

**Published:** 2015-03-12

**Authors:** A Ali, P Zhang, Y Liangfang, S Wenshe, H Wang, X Lin, Y Dai, X-h Feng, R Moses, D Wang, X Li, J Xiao

**Affiliations:** 1 Nortern Jiangsu People's Hospital (Medical College of Yangzhou University), Yangzhou, Jiangsu 225001, China; 2 Shanghai Key Laboratory of Regulatory Biology, Key Laboratory of Brain Functional Genomics (Ministry of Education), Shanghai Key Laboratory of Brain Functional Genomics, Institute of Biomedical Sciences, School of Life Sciences, East China Normal University, 500 Dongchuan Road, Shanghai 200241, China; 3 Department of Pathology, the Second Chengdu Municipal Hospital, Chengdu, Sichuan 610017, China; 4 Department of Molecular and Cellular Biology, Baylor College of Medicine, One Baylor Plaza, Houston, TX 77030, USA; 5 Department of Orthopedic Oncology, Changzheng Hospital, The Second Military Medical University, 415 Fengyang Road, Shanghai 200003, China

## Abstract

Inhibition of tumor suppressive signaling is linked to cancer progression, metastasis and epithelial–mesenchymal transition (EMT). Transforming growth factor*-β*1 (TGF-*β)*/Smad signaling plays an important role in tumor suppression. Kruppel-like-factor 17 (KLF17) is a negative regulator of metastasis and EMT. However, underlying mechanisms leading to tumor suppressive and anti-metastatic function of KLF17 still remains unknown. Here, we show that KLF17 plays an integral role in potentiating TGF-*β*/Smad signaling via Smad3-dependent pathway to suppress tumor progression. Intriguingly, TGF-*β*/Smad3 signaling induces KLF17 expression, generating a positive feedback loop. TGF-*β*/Smad3–KLF17 loop is critical for anti-metastasis and tumor inhibition in cancer cells. Mechanistically, silencing KLF17 reduced Smad3–DNA complex formation on Smad binding element (SBE) and affects the expression of TGF-*β*/Smad target genes. Moreover, KLF17 alters Smad3 binding pattern on chromatin. KLF17 regulates TGF-*β* target genes that are Smad3-dependent. Smad3 and KLF17 physically interact with each other via KLF17 responsive elements/SBE region. Intriguingly, TGF-*β* stimulates the recruitment of KLF17 on chromatin to subsets of metastasis-associated genes. Functionally, depletion of KLF17 enhanced tumorigenic features in cancer cells. KLF17 is critical for full cytostatic function of TGF-*β*/Smad signaling. Clinically, KLF17 expression significantly decreases during advance HCC. KLF17 shows positive correlation with Smad3 levels in cancer samples. Our data shows that enhance KLF17 activity has important therapeutic implications for targeted-therapies aimed at TGF-*β*/Smad3 pathway. These findings define novel mechanism by which TGF-*β*/Smad–KLF17 pathway mutually affect each other during cancer metastasis, provide a new model of regulation of TGF-*β*/Smad signaling by KLF17 and defines new insights into anti-metastatic function of KLF17.

Liver cancer represents fifth most common solid tumor among the different cancers. In males, Liver cancer is the second leading cause of cancer death and in females it is the sixth most common cause of cancer death.^1^ Among different liver cancers types, human hepatocellular carcinoma (HCC) is the most critical and represents about 70–85% of the total liver cancer burden. During HCC high frequency of metastasis, fast tumor progression, recurrence and poor survival rate remain a main challenge for HCC treatment.^1, 2^ To date, the exact mechanisms and biology of HCC remains poorly understood which hindered the development of new chemotherapeutic targets. To investigate the mechanisms involved in HCC metastasis and recurrence might be helpful in the development of novel chemotherapeutic targets.^2, 3^ Studies have shown that crosstalk and co-operation among different signaling, tumor-suppressor and oncogenic proteins play a crucial role in the epithelial–mesenchymal transition (EMT) and metastasis of cancer.^3^ Among these molecules and signaling Kruppel-like-factor 17 (KLF17) has been shown to have a critical impact on metastasis and cancer progression.

KLFs, which include KLF1–KLF17, are a subfamily of the mammalian Sp/KLF zinc-finger protein family. They have important roles in transcription by binding via their highly conserved DNA-binding domains (DBDs) or C-termini to related G/C and CACCC boxes of target genes.^4, 5, 6^ KLFs are implicated in tumor cell proliferation, invasion and metastasis. They function as transcriptional activators or suppressors as determined by regulatory proteins they bind to.^7, 8^ KLF17 gene encodes a suppressor of cancer cell metastasis.^9, 10, 11, 12^ KLF17 suppresses EMT and metastasis by directly binding to the promoter and inhibiting the transcription of Id1, which is a key regulator of tumorigenesis, EMT, angiogenesis, invasion and metastasis.^12, 13^ Forced expression of KLF17 in highly metastatic breast cancer cells inhibits the ability of these cells to metastasize to the lung.^11, 12^ Conversely, depletion of KLF17 enhances cancer cell migration and invasion.^9, 10, 11^ In addition, reduced expression of KLF17 is a predictor of metastasis in breast cancer, lung adenocarcinoma and HCC.^9, 12, 13^ Patients with decreased level of KLF17 have poor prognosis as exhibiting greater tumor size and advanced stages.^10, 11^

Transforming growth factor*-β*1 (TGF-*β*1) have a unique and pivotal role in homeostasis, wound healing, fibrosis, angiogenesis, carcinogenesis and differentiation of the cell.^14, 15, 16^ It is a potent inhibitor of epithelial cell proliferation, and induces apoptosis.^17, 18, 19^ TGF-*β*1 interacts with TGF-*β*RII, which in turn activates TGF-*β*RI. Smad2 and Smad3 are phosphorylated at the C terminus by activated TGF-*β*RI receptor and form heteromeric complexes with Smad4.^20, 21, 22^ Ultimately, the Smad2/3/4 complex translocates into the nucleus and binds to specific regulatory elements on target genes.^23, 24^ The role of TGF-*β* signaling as a tumor suppressor is best illustrated by the presence of inactivating mutations in genes encoding TGF-*β* receptors and Smads in human carcinomas, and by studies of tumor development in mouse models.^25, 26^ Mutations in *TGFBRII* are frequently found in colon cancers, gastric cancers and gliomas with microsatellite instability.^27, 28^ Mutations of the Smad2/3/4 encoding gene sequences have been detected in several carcinomas.^29^ TGF-*β* dependent transcription can have cross-talks with other signaling pathways through physical interactions and functional co-operativity between Smads and other transcription factors.^30, 31, 32^

Here, in this study we address the issue that how KLF17 control cancer progression and metastasis in cancer cells. We found that KLF17 potentiates TGF-*β*/Smad-dependent signaling to inhibit tumor formation. In search for the molecular mechanisms, we identified that KLF17 has an integral role in potentiating TGF-*β*/Smad3-dependent signaling during tumorigenesis. Intriguingly, TGF-*β*/Smad3 signaling and KLF17 forms a novel positive-feedback loop. KLF17 is important for the full transcriptional activity of TGF-*β*/Smad3 signaling. Knockdown of KLF17 decreased the binding of activated Smad3 to TGF-*β*/Smad target gene promoters. Importantly, this novel crosstalk between KLF17 and Smad3 occurs on a subset of metastasis-associated genes. KLF17 and Smad3 physically interact with each other via KLF17 responsive elements (KLF17RE)/smad binding elements (SBE) region. Thus, TGF-*β*/Smad3–KLF17 loop is critical for anti-metastasis and tumor inhibition in cancer cells.

## Results

### TGF-*β* enhances KLF17 expression via Smad3

**Figure 1:**
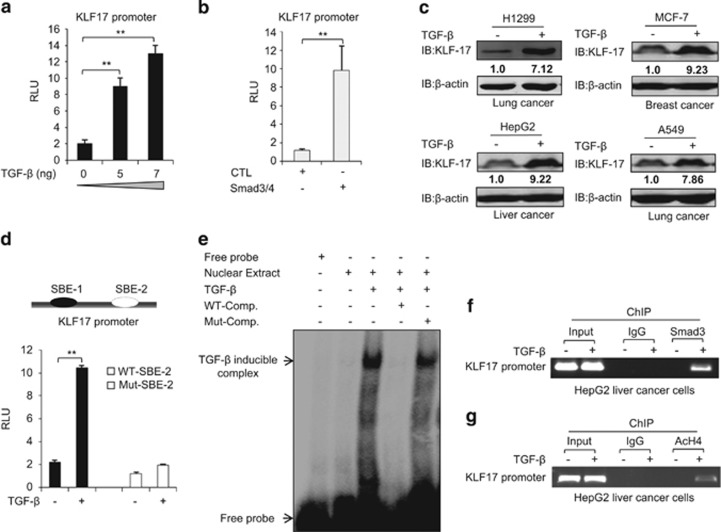
Upregulation of KLF17 expression by TGF-*β*-SMAD3 signaling (**a**) HepG2 cells were transfected with a KLF17 reporter construct (2 *μ*g), treated with different doses of TGF-*β* for 24 h prior to lysis, and analyzed for luciferase activity. Data are representative of three independent experiments (mean±S.D.). (Two-tailed Student's *t*-test, *P<0.05, **P<0.005). (**b**) HepG2 cells were co-transfected with a KLF17 reporter construct (2 *μ*g) in combination with Smad3/4 (100 ng) expression plasmids for 24 h, and then analyzed for luciferase activity. The average was calculated based on three independent experiments with mean±S.D. (Two-tailed Student's *t*-test, *P<0.05, **P<0.005). (**c**) A549, HepG2, MCF-7 and H1299 cells were treated with 5 ng/ml TGF-*β* and analyzed by Western blot. (**d**) Schematic representation of putative SBE boxes in the KLF17 promoter. The white arrow indicates functional SBE in KLF17 promoter. HepG2 cells were transfected with wild-type (2 *μ*g) or mutated (2 *μ*g) SBE KLF17 luciferase reporter constructs. Cells were then left untreated or treated with 5 ng/ml TGF-*β* for 24 h, and luciferase activity was measured. Data are representative of three independent experiments with mean±S.D. (Two-tailed Student's *t*-test, **P<0.05). (**e**) EMSA was performed using nuclear extract from HepG2 cells treated with 5 ng/ml TGF-*β* for 24 h. About 2 *μ*g of nuclear extract protein were incubated with ^32^p-radiolabeled probe containing SBE box from KLF17 promoter. (**f**) HepG2 cells were treated with 5 ng/ml TGF-*β* for 24 h, and chromatin immunoprecipitation were performed with indicated antibodies. (**g**) HepG2 cells were treated with 5 ng/ml TGF-*β* for 24 h, and chromatin immunoprecipitation were performed with indicated antibodies

Using bioinformatics analysis, NCBI database, we found SBEs within KLF17 promoter. This finding prompted us to analyze the potential regulation of KLF17 by TGF-*β*. In a dose-dependent manner, TGF-*β* was able to induce the transcriptional activity of KLF17-luc reporter (Figure 1a and Supplementary Figure 1A). Ectopic expression of Smad3/4 also induced KLF17-luc activity (Figure 1b). TGF-*β* treatment also enhanced KLF17 protein levels in multiple cancer cell lines (Figure 1c). While, depletion of Smad3 reduced KLF17 expression in different cancer cells (Supplementary Figure 1B).

There are two potential SBE sites in KLF17 promoter. To determine the functional importance of these SBEs in mediating TGF-*β*-regulated KLF17 expression, we designed oligo probes containing each putative SBE and tested their ability to bind to purified Smad3 protein. We found that only SBE-2 bound strongly to Smad3 (Supplementary Figure 1C). Moreover, mutation of SBE-2 within the KLF17-luc reporter blunted its response to TGF-*β* induction, indicating that an activated Smad complex binds to SBE-2 to induce KLF17 transcription (Figure 1d). As expected, we observed a TGF-*β-*induced Smad complex binding to SBE-2 in an electrophoretic mobility shift assay (EMSA) using nuclear extract (Figure 1e). Furthermore, we detected the enrichment of Smad3 and AcH4 (acetylated histone 4) in TGF-*β* responsive (SBE-2) region on KLF17 promoter by chromatin immnuoprecipitation (ChIP) assay (Figures 1f and g). These results explain some of the published results that TGF-*β* can induce the expression of KLF17 target genes.^33^

### KLF17 regulates TGF-*β* target genes by modulating Smad3–DNA complex formation

**Figure 2:**
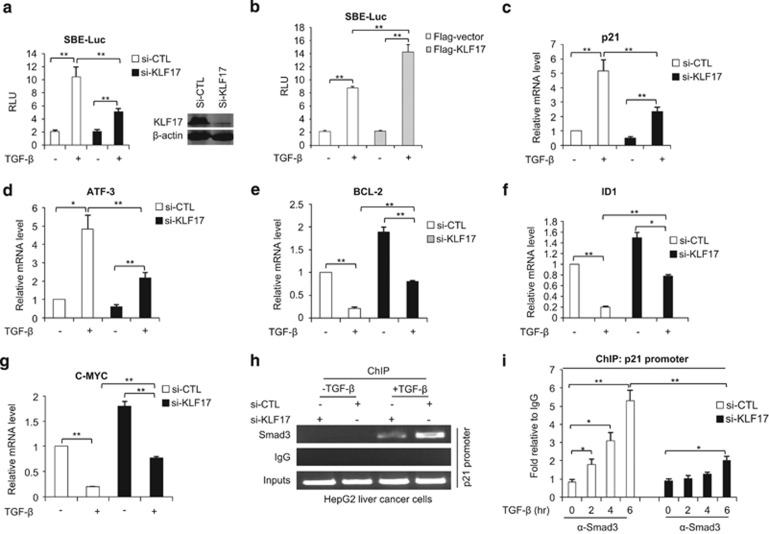
KLF17 is required for full transcriptional activity of TGF-*β*-SMAD pathway (**a**) HepG2 cells were transfected with control siRNA or siRNA targeting against KLF17 (20 nM) for 48 h and then transfected with SBE reporter construct (2 *μ*g), and left untreated or treated with 5 ng/ml TGF-*β* for 24 h prior to lysis, and analyzed for luciferase activity. Data are representative of three independent experiments (mean±S.D.). (Two-tailed Student's *t*-test, *P<0.05, **P<0.005). (**b**) HepG2 cells were co-transfected with vector encoding KLF17 and SBE luciferase reporter for 24 h prior to lysis, and analyzed for luciferase activity. Data are representative of three independent experiments (mean±S.D.). (Two-tailed Student's *t*-test, *P<0.05, **P<0.005). (**c**–**g**) HepG2 cells were transfected with control siRNA or siRNA targeting against KLF17 (20 nM) for 48 h, and then left untreated or treated with 5 ng/ml TGF-*β* for 14 h, and analyzed by RT-PCR analysis. Data are representative of three independent experiments (mean±S.D.). (Two-tailed Student's *t*-test, *P<0.05, **P<0.005). (**h**) HepG2 cells were transfected with control siRNA or siRNA targeting against KLF17 (20 nM) for 48 h, and then left untreated or treated with 5 ng/ml TGF-*β* for indicated time points and analyzed by ChIP assay with indicated antibodies. (**i**) HepG2 cells were transfected with control siRNA or siRNA targeting against KLF17 (20 nM) for 48 h, and then left untreated or treated with 5 ng/ml TGF-*β* with indicated time points and analyzed by ChIP assay with Smad3 antibody. Data are representative of three independent experiments (mean±S.D.). (Two-tailed Student's *t*-test, *P<0.05, **P<0.005)

Previous publications indicate that some of the genes directly regulated by TGF-*β* can also be targets of KLF17.^34, 35^ To investigate the role of KLF17 in TGF-*β*/Smad-mediated signaling, we manipulated expression of KLF17 by RNA interference (RNAi) in cancer cell lines and tested TGF-*β* responsive SBE-containing luciferase reporter (SBE-Luc) activities. We found that SBE-Luc transcriptional activity was higher in control cells than in KLF17 knockdown cells following TGF-*β* treatment (Figure 2a and Supplementary Figure 2A). In contrast overexpression of KLF17 further enhanced SBE-Luc transcriptional activity in TGF-*β*-treated cells (Figure 2b). On sufficient depletion of KLF17 by RNAi (Figure 2a, right panel and Supplementary Figure 2B), expression of p21, ATF-3, BCL-2, ID1 and C-Myc genes that are regulated by TGF-*β*/Smad signaling showed attenuated response to TGF-*β* treatment (Figures 2c–g and Supplementary Figures 2C and D).

To investigate the molecular mechanism underlying KLF17 regulated expression of TGF-*β* target genes, we performed ChIP analysis of the p21 promoter, a well-known target of TGF-*β*/Smad3 signaling. We observed a decreased recruitment of Smad3 to p21 promoter in KLF17 knockdown cells (Figure 2h). Furthermore, quantitative-ChIP analysis of p21 promoter in response to TGF-*β* with indicated time period obtained similar results (Figure 2i). These data indicate that KLF17 regulates TGF-*β* target genes most likely through regulating Smad3.

### KLF17 induces Smad3 to generate a positive feedback loop

**Figure 3:**
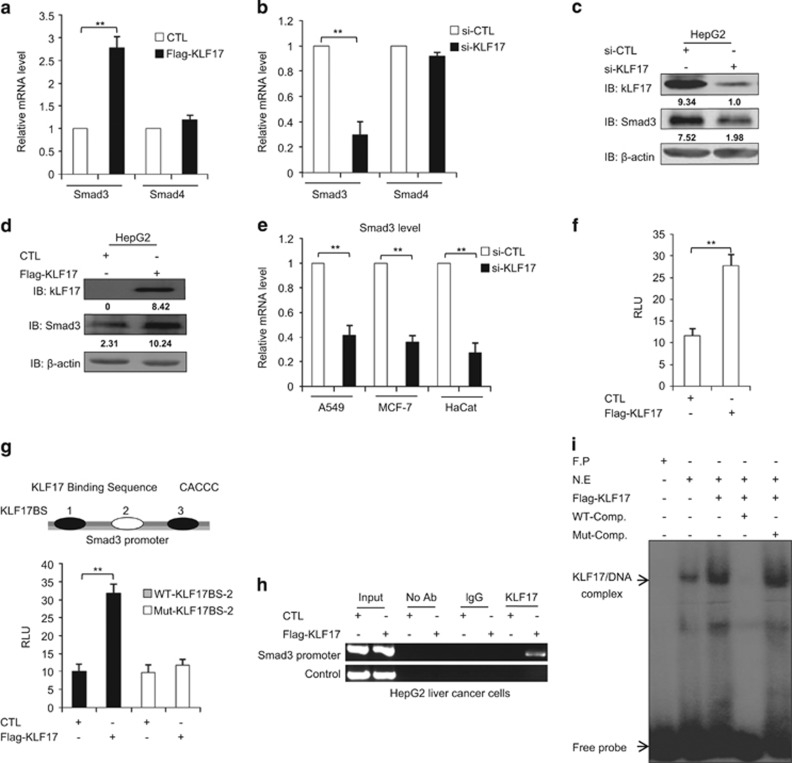
KLF17 promotes SMAD3 transcription (**a**) HepG2 cells were transfected with either flag vector or expression plasmid encoding KLF17 for 24 h and subjected to RT-PCR analysis. Data are representative of three independent experiments (mean±S.D.). (Two-tailed Student's *t*-test, *P<0.05, **P<0.005). (**b**) HepG2 cells were transfected with control siRNA or siRNA targeting against KLF17 (20 nM) for 48 h, and analyzed by RT-PCR. Data are representative of three independent experiments (mean±S.D.). (Two-tailed Student's *t*-test, *P<0.05, **P<0.005). (**c**) HepG2 cells were transfected with control siRNA or siRNA targeting against KLF17 (20 nM) for 72 h, and analyzed by immunoblot with indicated antibodies. (**d**) HepG2 cells were transfected with either flag vector or expression plasmid encoding KLF17 for 24 h and subjected to immunoblot analysis with indicated antibodies. (**e**) Cancer cells were transfected with control siRNA or siRNA targeting against KLF17 (20 nM) for 48 h, and analyzed by RT-PCR. The average was calculated based on three independent experiments with mean±S.D. (Two-tailed Student's *t*-test, *P<0.05, **P<0.005). (**f**) HepG2 cells were co-transfected with Smad3 reporter construct and expression plasmid encoding KLF17 for 24 h prior to lysis, and analyzed by luciferase assay. Data are representative of three independent experiments (mean±S.D.). (Two-tailed Student's *t*-test, *P<0.05, **P<0.005). (**g**) Schematic representation of putative KLF17BS boxes in Smad3 promoter. The white arrow indicates functional KLF17BS in Smad3 promoter. HepG2 cells were transfected with wild-type (2 *μ*g) or mutated (2 *μ*g) KLF17BS along with Smad3 luciferase reporter constructs for 24 h and then analyzed by luciferase assay. Data are representative of three independent experiments (mean±S.D.). (Two-tailed Student's *t*-test, *P<0.05, **P<0.005). (**h**) HepG2 cells were transfected with either flag vector or expression plasmid encoding KLF17 for 24 h and subjected to ChIP analysis with indicated antibodies. (**i**) HepG2 cells were transfected with either flag vector or expression plasmid encoding KLF17 for 24 h and subjected to EMSA analysis with radiolabel p^32^ probe corresponding to KLF17BS in Smad3 promoter

Next, we sought to address how KLF17 affects the Smad3-dependent signaling. We found that overexpression of KLF17 in HepG2 cells increased Smad3, but not Smad4 mRNA level (Figure 3a). On the contrary, silencing KLF17 in HepG2 cells reduced Smad3, but not Smad4 mRNA levels (Figure 3b). By Western blot, we verified our finding that KLF17 positively regulates Smad3 (Figures 3c and d). Knockdown of KLF17 expression in multiple cancer cell lines showed similar decrease in Smad3 mRNA levels (Figure 3e), substantiating that KLF17 induces Smad3 expression in multiple cells.

Given that KLF17 functions as a transcription factor to regulate its target gene expression at transcriptional level,^9, 10, 11^ we intend to determine if Smad3 is a direct target gene of KLF17. By co-expressing a Smad3 luciferase reporter (Smad3-Luc) along with KLF17, we found that KLF17 was able to induce Smad3-Luc activity (Figure 3f). KLF17 has been known to bind to CACCC box sequences.^6, 7, 8^ We found three KLF17 responsive elements in the Smad3 promoter region (Figure 3g). We designed oligo probes corresponding to these sites, and detected the binding of KLF17 to KLF17RE-2, but not to the others sites by EMSA (Supplementary Figure 3A). Furthermore, we mutated the KLF17RE-2 in the Smad3-Luc reporter and found that KLF17 was unable to induce Smad3 activity, suggesting that the KLF17RE-2 site is critical for regulation of Smad3 by KLF17 (Figure 3g). Consistently, *in vivo* recruitment of KLF17 to the Smad3 promoter by ChIP analysis (Figure 3h) and *in vitro* binding of KLF17 to KLF17RE-2 elements by EMSA analysis (Figure 3i) all demonstrated that KLF17 directly regulates transcription of Smad3.

### KLF17 regulates a panel of TGF-*β*/Smad3-dependent target genes

**Figure 4:**
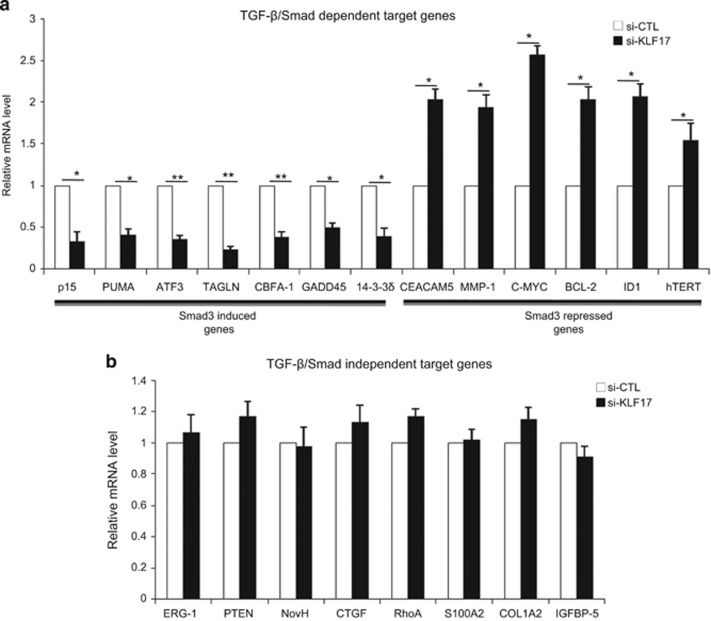
KLF17 regulates a panel of TGF-*β*-SMAD3-dependent target genes. (**a** and **b**) HepG2 cells were transfected with control siRNA or siRNA targeting against KLF17 (20 nM) for 48 h, and analyzed by RT-PCR. Data are representative of three independent experiments (mean±S.D.). (Two-tailed Student's *t*-test, *P<0.05, **P<0.005)

Based on the above findings, we hypothesize that KLF17 may have broader impact on the transcriptional regulation of TGF-*β*/Smad-dependent target genes. We thus examined the levels of several TGF-*β*/Smad-dependent target genes in KLF17 knockdown cells and control cells. Using Real time-PCR (RT-PCR) approach, we observed changes in the expression of several TGF-*β*/Smad-dependent target genes between control and KLF17 knockdown cells (Figure 4a), suggesting that KLF17 indeed influences the transcriptional program of the TGF-*β*/Smad pathway. Importantly, genes upregulated by Smad3/4 in response to TGF-*β*, including *p15, PUMA, ATF-3, TGLN, CBFA-1 and 14-3-3δ*,^36, 37, 38^ were repressed in the absence of KLF17 (Figure 4a). In contrast, genes repressed by Smad3/4, such as *CEACAM-5, MMP-1, C-MYC, BCL-2, ID1 and hTERT,*^36, 39, 40^ were de-repressed when KLF17 was depleted, indicating a positive regulation of Smad3 by KLF17 (Figure 4a).

Next, we sought to address the impact of KLF17 on regulation of TGF-*β* Smad-independent target genes. We selected a list of genes, which are regulated by TGF-*β* signaling in Smad-independent manner such as *ERG-1, PTEN, NovH, CTGF, RhoA, S100A2, COL1A2 and IGFBP-5*. Intriguingly, we observed no significant changes in the expression of these genes on depletion of KLF17 (Figure 4b). In conclusion, these data suggest that KLF17 has the ability to regulate TGF-*β*/Smad-dependent target genes, but not those independent of Smads.

### Joint regulation of metastasis-associated genes by KLF17–Smad3 signaling

**Figure 5:**
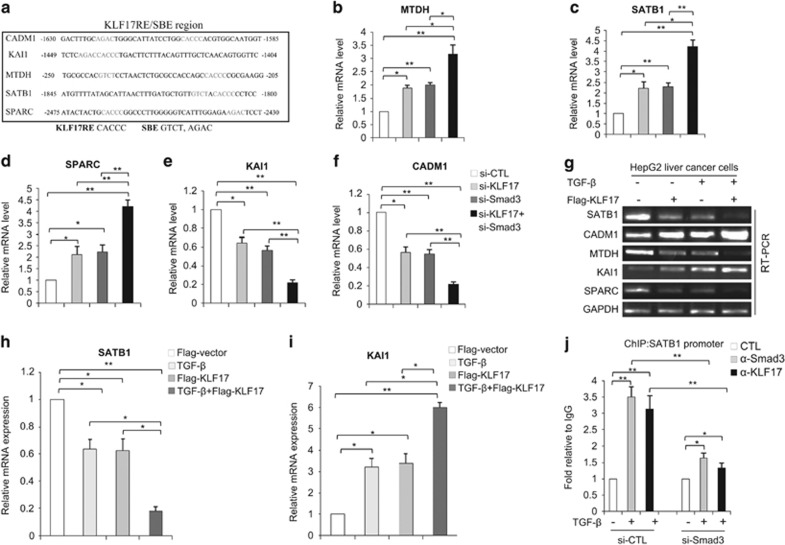
Cross-talk between KLF17 and SMAD3 on a subset of metastasis-associated genes (**a**) Schematic representation of adjacent KLF17BS and SBE region on a subset of metastasis-associated genes within 50 bp. (**b**–**f**) HepG2 cells were transfected with siRNAs targeting against Smad3 (20 nM) and KLF17 (20 nM) either independently or in combination for 48 h and then subjected to RT-PCR analysis. Data are representative of three independent experiments with mean±S.D. (two-tailed Student's *t*-test, *P<0.05, **P<0.005). (**g**–**i**) HepG2 cells were transfected with an expression plasmid encoding KLF17 and left untreated or treated with 5 ng/ml TGF-*β*, and subjected to semi-quantitative RT-PCR. (**j**) HepG2 cells were transfected with siRNA targeting against Smad3 (20 nM) and then left untreated or treated with 5 ng/ml TGF-*β* for 24 h and subjected to ChIP analysis with indicated antibodies. Data are representative of three independent experiments with mean±S.D. (two-tailed Student's *t*-test, *P<0.05, **P<0.005)

Previous studies have shown transcriptional cross-talks between TGF-*β*/Smad signaling and other pathways. These cross-talks require adjacent binding sites for Smad and other transcription factors.^30, 31, 32^ Intriguingly, we found adjacent binding sites (within 50 bp) for KLF17 and SBE on a subset of metastasis-associated genes including MTDH, sequence-binding protein-1(SATB1), SPARC, KAI1 and CADM1 (Figure 5a). These sites were termed KLF17RE/SBE regions. Co-silencing of KLF17 and Smad3 resulted in significant upregulation of MTDH, SATB1 and SPARC genes and downregulation of KAI1 and CADM1 genes in comparison with independent depletion of either KLF17 or Smad3 (Figures 5b–f), suggesting that cross-talks between KLF17 and Smad3 occurs on these genes. Next, HepG2 cells were transfected with a control vector or a KLF17 expression plasmid in the presence or absence of TGF-*β*. RT-PCR analysis was performed and showed that the combined effect (KLF17+TGF-*β*) was much stronger than that of KLF17 transfection or TGF-*β* treatment alone (Figures 5g–i).

Next, we silenced Smad3 expression in HepG2 cells followed by ChIP analysis. The results showed decreased recruitment of both Smad3 and KLF17 to special AT-rich SATB1 promoter in TGF-*β*-treated cells depleted Smad3 (Figure 5j and Supplementary Figure 4A), consistent with that activated Smad3 cooperates with KLF17 via promoter region in response to TGF-*β*.

To further validate the co-operation between the Smad3 and KLF17 proteins in the KLF17RE/SBE regulatory region, we performed EMSA. Incubation of nuclear extract with a probe corresponding to the KLF17RE/SBE region resulted in formation of a putative KLF17–Smad–DNA complex (Supplementary Fig. 4B, Lane2), which was further enhanced in the presence of TGF-*β* (Supplementary Fig. 4B, Lane3). Addition of Smad3, Smad4 or KLF17 antibody alone significantly attenuated the formation of this complex (Supplementary Fig. 4B Lane4, 5 and 6), suggesting that TGF-*β* stimulates the formation of KLF17–Smad–DNA complex in this region. Finally, addition of both KLF17 and Smad3 antibodies nearly abolished the formation of this complex (Supplementary Fig. 4B, Lane7), substantiating that these complexes contained both KLF17 and Smad3 proteins. Taken together, these data indicate that Smad3 and KLF17 cooperate with each other via the KLF17RE/SBE region, and simultaneously control the expression of these genes.

### KLF17 inhibits proliferation in cancer cells

**Figure 6:**
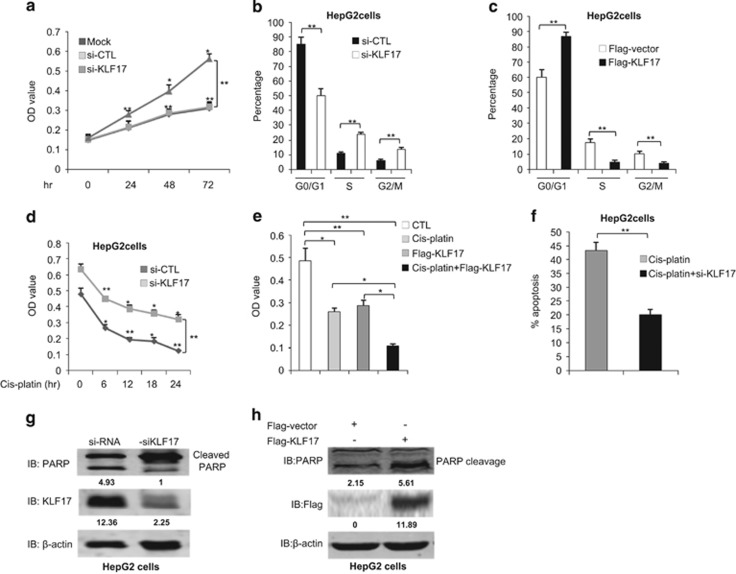
Knockdown of KLF17 affects multiple cellular functions. (**a)** HepG2 cells were transfected with control siRNA or siRNA targeting against KLF17 (20 nM) for indicated time points and subjected to MTT analysis. Data are representative of three independent experiments with mean±S.D. (two-tailed Student's *t*-test, *P<0.05, **P<0.005). (Two way ANOVA, **P<0.005). (**b**) HepG2 cells were transfected with control siRNA or siRNA targeting against KLF17 (20 nM) for 72 h and subjected to FACS analysis. Data are representative of three independent experiments with mean±S.D. (two-tailed Student's *t*-test, *P<0.05, **P<0.005). (**c**) HepG2 cells were transfected with either flag vector or expression plasmid encoding KLF17 for 24 h and subjected to FACS analysis. Data are representative of three independent experiments (mean±S.D.). (two-tailed Student's *t*-test, *P<0.05, **P<0.005). (**d**) HepG2 cells were transfected with control siRNA or siRNA targeting against KLF17 (20 nM) for 48 h and then left untreated or treated with cisplatin for indicated time points and subjected to MTT analysis. Data are representative of three independent experiments with mean±S.D. (two-tailed Student's *t*-test, *P<0.05, **P<0.005). (two way ANOVA, **P<0.005). (**e**) HepG2 cells were transfected with either flag vector or an expression plasmid encoding KLF17 and left untreated or treated with cisplatin for 12 h and subjected to MTT analysis. Data are representative of three independent experiments (mean±S.D.). (two-tailed Student's *t*-test, *P<0.05, **P<0.005). (**f**) HepG2 cells were transfected with control siRNA or siRNA targeting against KLF17 (20 nM) and then left untreated or treated with cisplatin (5 *μ*M) and subjected to FACS analysis to determine the apoptotic level. Data are representative of three independent experiments with mean±S.D. (Two-tailed Student's *t*-test, *P<0.05, **P<0.005). (**g**) HepG2 cells were transfected with control siRNA or siRNA targeting against KLF17 (20 nM) for 72 h and subjected to immunoblot analysis with indicated antibodies. (**h**) HepG2 cells were transfected with either flag vector or expression plasmid encoding KLF17 for 24 h and subjected to immunoblot analysis with indicated antibodies

To investigate the physiological function of KLF17 on cytostatic behavior in cancer cells, we measured cell growth capacity with manipulated expression of KLF17. 3-(4,5-dimethylthiazol-2-yl)-2,5-diphenyltetrazolium bromide (MTT) assays showed that KLF17-depleted cell lines were much more proliferated as compared with control cells (Figure 6a and Supplementary Figure 6B), indicating that KLF17 inhibits cell growth. Moreover, depletion of KLF17 attenuated DNA content in GO/G1 phase, enhanced S (DNA synthesis) and G2/M phases (Figure 6b). In contrast, overexpression of KLF17 induced the cell cycle resting phase GO/G1, decreased S and G2/M phases to reduce DNA synthesis (Figure 6c), indicating a dramatic impact on cell cycle progression.

Regulation of cell growth by KLF17 may have significant impact on cancer chemotherapy. To test this, we treated the control HepG2 or KLF17 knockdown cells with cisplatin, an anti-cancer drug, followed by MTT assays. Compared with control cells, KLF17-depleted cell lines were less sensitive to anti-cancer drug treatment (Figure 6d and Supplementary Figure 5A). Conversely, we examined the effect of KLF7 overexpression on cell response to cisplatin treatment. Either overexpression of KLF17 or cisplatin treatment reduced cell proliferation, while combination of KLF17 overexpression and cisplatin application further inhibited cell growth (Figure 6e). To understand the effect of KLF17 on cellular sensitivity to cisplatin-induced cell death, we knocked down KLF17 in HepG2 cells followed by cell cycle analysis of sub-G0/1 population, in the presence or absence of cisplatin. FACS analysis showed that HepG2 cells were less susceptible to cisplatin-induced apoptosis when KLF17 was depleted (Figure 6f), suggesting that KLF17 may increase cellular sensitivity to anti-cancer chemotherapy. Furthermore, we examined the level of Poly (ADP-ribose) polymerase (PARP) cleavage. Knockdown of KLF17 decreased apoptotic levels as indicated by reduced PARP cleavage (an apoptotic marker) (Figure 6g). In contrast, overexpression of KLF17 enhanced apoptotic level in HepG2 liver cancer cells (Figure 6h).

### KLF17 is critical for effective TGF-*β*/Smad3 function

**Figure 7:**
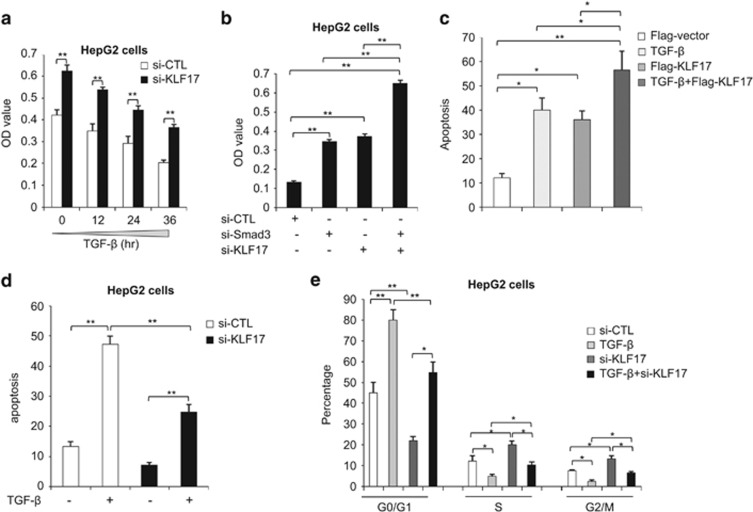
KLF17 is important for tumor suppressive function of TGF-*β*/SMAD3 pathway. (**a**) HepG2 cells were transfected with control siRNA or siRNA targeting against KLF17 (20 nM) for 48 h and then left untreated or treated with 5 ng/ml TGF-*β* for indicated time points and MTT assay was performed. Data are representative of three independent experiments with mean±S.D. (two-tailed Student's *t*-test, *P<0.05, **P<0.005). (**b**) HepG2 cells were transfected with siRNAs targeting against Smad3 (20 nM) and KLF17 (20 nM) either independently or in combination for 72 h and then subjected to MTT analysis. Data are representative of three independent experiments with mean±S.D. (two-tailed Student's *t*-test, *P<0.05, **P<0.005). (**c**) HepG2 cells were transfected either with flag vector or expression plasmid encoding KLF17 and then left untreated or treated with 5 ng/ml TGF-*β* and subjected to FACS analysis to detect apoptotic level. Data are representative of three independent experiments (mean±S.D.). (Two-tailed Student's *t*-test, *P<0.05, **P<0.005). (**d**) HepG2 cells were transfected with control siRNA or siRNA targeting against KLF17 (20 nM) for 48 h and then left untreated or treated with 5 ng/ml TGF-*β* for 24 h and FACS analysis was performed to detect the apoptotic level. Data are representative of three independent experiments with mean±S.D. (two-tailed Student's *t*-test, *P<0.05, **P<0.005). (**e**) HepG2 cells were transfected with control siRNA or siRNA targeting against KLF17 (20 nM) for 48 h and then left untreated or treated with 5 ng/ml TGF-*β* for 24 h and FACS analysis was performed to detect the cell cycle progression. Data are representative of three independent experiments with mean±S.D. (two-tailed Student's *t*-test, *P<0.05, **P<0.005)

Next, we aimed to address the impact of KLF17 regulation on cellular response to TGF-*β* activation. HepG2 cells treated with a control or a specific small interference RNA (siRNA) against KLF17 were measured for growth inhibition by TGF-*β*. KLF17-depleted cells were less sensitive to TGF-*β* treatment and more proliferative in comparison with control cells (Figure 7a). Furthermore, knockdown of KLF17 or Smad3 alone almost equally enhanced cell growth (Figure 7b). Co-silencing of KLF17 and Smad3 further induced cell proliferation (Figure 7b), suggesting that KLF17 might have interplay with Smad3 signaling in the inhibition of cell growth.

TGF-*β* signaling is known to regulate cellular apoptosis and cell cycle progression,^16, 17, 18^ therefore we tested the influence of KLF17 on these TGF-*β*-dependent functions. Knockdown or overexpression of KLF17 affected the apoptotic levels of HepG2 cells in response to TGF-*β* (Figures 7c and d), suggesting a dramatic effect of KLF17 on TGF-*β*-mediated control over cell death. Moreover, depletion of KLF17 attenuated the effect of TGF-*β* on cell cycle progression, showing enhanced S and G2/M phases when compared with controls (TGF-*β* treatment alone) with combination of TGF-*β* and siKLF17 treatments (Figure 7e).

### KLF17 expression correlates with Smad3 in cancer cells

**Figure 8:**
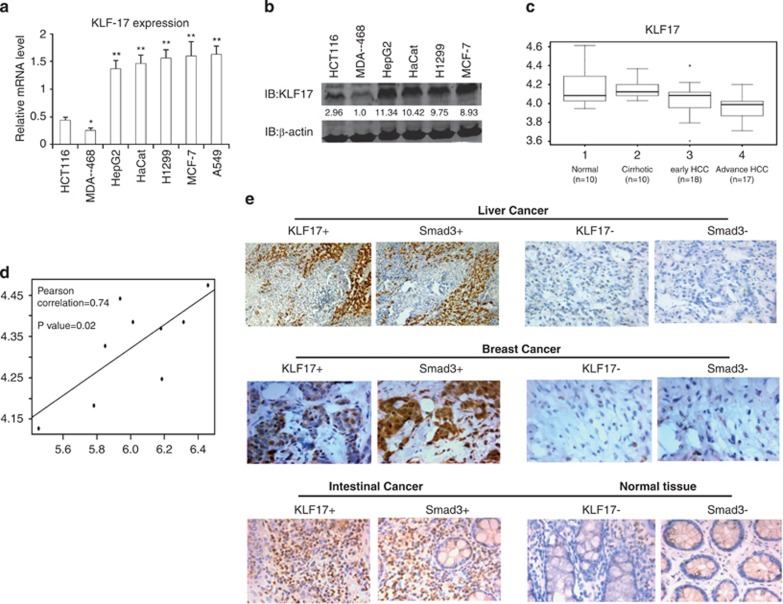
Correlation of SMAD3 and KLF17 expression in multiple cancer cells (**a**) KLF17 expression in multiple cancer cell lines was detected by RT-PCR. Data are representative of three independent experiments with mean±S.D. (two-tailed Student's *t*-test, *P<0.05, **P<0.005). (**b**) KLF17 expression in multiple cancer cell lines was detected by immunoblot analysis. (**c**) Inverse correlation between KLF17 expression and tumor stages in human liver cancer. Data and statistics were obtained from GEO data base. (**d**) Bioinformatics analysis of Lung cancer data set in which Smad3 expression is positively correlated with KLF17. Pearson's correlation coefficient was used as a measure of correlation between Smad3 and KLF17. Pearson's correlation analysis was conducted using ‘R program' on data set obtained from http://www.ncbi.nlm.nih.gov/geo/. (**e**) IHC analysis between Smad3 and KLF17 in liver (*n*=2), breast (*n*=9) and intestinal cancer (*n*=6), respectively

Next, we examined KLF17 RNA expression in several human cancer cell lines. In contrast to Smad-inactive (HCT116) and Smad4 null (MDA-MB-468) cancer cell lines,^41^ we observed higher levels of KLF17 in Smad-expressing cells such as HepG2, MCF-7, HaCaT and H1299 (Figure 8a). We obtained similar results for their protein levels by Western blot analysis (Figure 8b).

To determine the physiopathological relevance of KLF17 with human cancer development, we analyzed clinical data to see whether KLF17 expression correlated with disease progression in human HCC. A comparison of KLF17 expression levels among normal tissues and HCC cancer samples at various stages was performed according to the Oncomine database www.oncomine.org, which provides published data sets on gene expression in cancers. Results from GSE6764 data set showed that KLF17 is significantly downregulated during cirrhosis and HCC progression (Figure 8c). Importantly, we observed an inverse correlation between KLF17 expression and HCC tumor grades (Figure 8c), suggesting that decreased expression of KL17 may be associated with poor prognosis.

Moreover, we performed computational analysis to understand the correlation between KLF17 and Smad3 expressions in lung cancer data sets and found that these two tumor-suppressor proteins had strong positive correlation with each other in lung cancers (Figure 8d and Supplementary Figure 6), consistent with the molecular basis of regulatory features in KLF17 and Smad3 identified above.

To further substantiate the biological relevance of our findings about mutual regulation of KLF17 and Smad3 in tumor development, we evaluated the correlation between Smad3 and KLF17 expression by IHC analysis using liver, breast and intestinal cancer tissues in a total of 17 cases with adjacent 'non-tumor' controls. Interestingly, KLF17 expression was strongly correlated with Smad3 level in all tested cancer samples except 1 (Figure 8e, labeled as KLF17+ and Smad3+, and Supplementary Fig. 7). Among the highly correlated cancer samples, two displayed low levels of KLF17 and Smad3 expression (Figure 8e, KLF17− and Smad3−).

## Discussion

Cancer metastasis is a complex process that involves numerous critical and influential molecules/proteins. Among these important factors KLF17 has ability to inhibit metastasis and EMT. However, the molecular details that how KLF17 control cancer metastasis remains unclear. In this study, we provide new insights into the anti-metastasis and tumor suppressive function of KLF17. We indentified that KLF17 has a key role to potentiate TGF-*β*/Smad-dependent pathway to suppress cancer progression. Our finding, suggest that KLF17 is one of the key regulator of TGF-*β*/Smad-dependent pathway. Our results indicate that loss of KLF17 impairs tumor suppressive function of TGF-*β*/Smad-dependent pathway. Moreover, in this report, we investigated a previously unknown regulatory mechanism for the regulation of KLF17 by TGF-*β*/Smad3 signaling in cancer cells. It is generally believed that the Smad-dependent pathway is involved in TGF-*β* tumor suppressive functions at an early stage of cancer development, whereas activation of Smad-independent pathways is coupled with the loss of tumor-suppressor function of TGF-*β*, which is important for its pro-oncogenic effects.^30^ TGF-*β* enhances the protein level of KLF17 in multiple cancer cell lines. Thus, induction of KLF17 by TGF-*β*/Smad3 may be one of the mechanisms to fight against cancer development.

**Figure 9:**
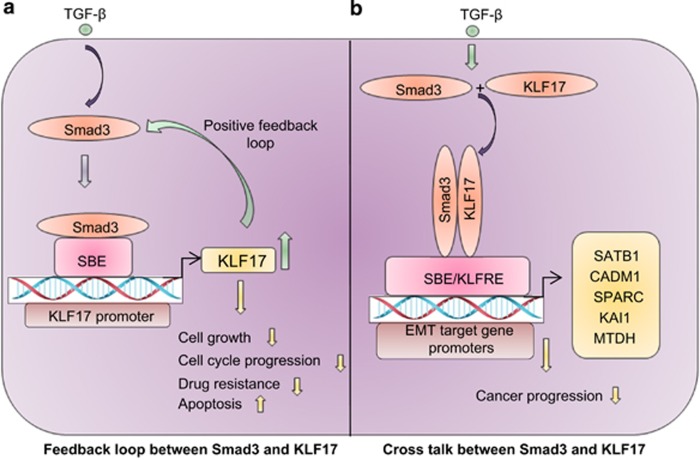
A simplified model depicting (**a**) Novel positive feedback loop between TGF-*β*-SMAD3 and KLF17 in cancer cells. (**b**) Novel cross-talk between SMAD3- KLF17 pathways on a subset of metastasis-associated genes via KLF17RE/SBE region in cancer cells

We show that KLF17 is required for the full transcriptional activity of TGF-*β*/Smad3 signaling. In our search for the molecular mechanisms by which KLF17 affects the TGF-*β*/Smad signaling, we have found that KLF17 induces Smad3 transcription in cultured cells, indicating novel autoregulatory positive feedback loop with Smad3 (Figure 9). Due to this regulation, depletion of KLF17 decreases the recruitment of Smad3 to its target gene promoters by reduced SBE/DNA–Smad complex formation. In fact, activated Smad3 and KLF17 mutually affect each other transcriptionally to inhibit cell proliferative effects. Thus, a possible significance of this loop is that both proteins can cooperatively enhance the transcription of target genes in cancer cells to overcome cancer progression.

We also found a novel cross-talk between Smad3 and KLF17 on a subset of metastasis-associated genes in HepG2 cells. The combinational loss of Smad3 and KLF17 function can significantly change the expression of these metastasis genes. Both KLF17 and Smad3 interact with the KLF17RE/SBE region and form a transcription complex in a TGF-*β*-dependent manner. Thus, combinatorial control of gene expression by KLF17–Smad3 pathways provides a new tier in regulation of TGF-*β* responsive genes. Our mechanistic study provides an insight to the well-known observation that both KLF17 and TGF-*β*/Smad3 can regulate ID1 and impinge on cancer cell metastasis.^9^

In addition to cytostatic effect, KLF17 is also critical for tumor-suppressor function of TGF-*β* in regulating cell cycle and cell death. Depletion of KLF17 attenuates cellular response to TGF-*β*-mediated inhibition of cell cycle progression and induction of cell apoptosis, which may contribute to enhanced chemoresistance in cultured cell. Furthermore, we show that KLF17 increases the sensitivity of cancer cells response to anti-cancer drugs. All our results highlight a tight association between KLF17 and TGF-*β*/Smad tumor suppressive functions and are endorsed by other publications that reduced KLF17 is potentially associated with advanced cancer progression.^6, 7, 8, 9^ Thus, cancer patients with higher levels of KLF17 may benefit from treatment with chemotherapeutic agents.

Finally, our study indicates that targeting KLF17 for cancer therapy may be beneficial for some human tumors having abnormal Smad3 proteins. For instance, Smad3 inactivation is known to accelerate cancer metastasis.^42, 43, 44, 45, 46^ Given the complicated roles of TGF-*β* pathway in the regulation of cancer metastasis,^47, 48^ it remains unclear whether KLF17 may cooperatively or discordantly affect TGF-*β* signaling in different tumor metastasis. These findings are the first evidence for the cross-talks over TGF-*β*–Smad3–KLF17 pathways. Even though growing evidence suggests that KLF17 is involved in the inhibition of metastasis and EMT, great efforts are still needed to further understand KLF17 regulation during cancer progression.

## Materials and Methods

### Plasmids and transfection

pcDNA3.1-KLF17 was constructed. While, pRK5-Smad3 and pRK5-Smad4 were kindly provided by Dr. Xin Hua Feng, Baylor College of Medicine. HepG2 cell lines were transfected with Lipofectamine 2000 following manufacturer's protocol (Invitrogen, Life Technologies, Carlsbad, CA, USA).

### Antibodies

Following antibodies were used in Western Blot, EMSA and ChIP experiments: anti-Smad3 (Cell Signaling Technology, Danvers, MA, USA), anti-Smad4 (Santa Cruz Biotechnology, Dallas, TX, USA), anti-*β* actin (Santa Cruz Biotechnology), anti-AcH4 (Millipore, Temecula, CA, USA), anti-KLF17 (Abcam, Cambridge, UK).

### Cell culture and treatments

HaCaT, MCF-7, HepG2 and A549 were purchased from American Type Culture Collection (ATCC, Manassas, VA, USA). For cells treatments, we used 5 ng/ml TGF-*β*1 (R&D Systems, Minneapolis, MN, USA) and 5 *μ*g/ml of cisplatin (Sigma-Aldrich, St. Louis, MO, USA).

### Electrophoretic mobility shift assay

EMSA was performed as described previously.^30^ Briefly, EMSA was performed with ^32^P-radiolabeled probes. About 2 *μ*g of nuclear extract or different concentration of purified proteins was incubated with ^32^P-radiolabeled-probes in 20 *μ*l of EMSA reaction buffer (2 *μ*g of poly (dI-dC), 20 mM HEPES (pH 7.9), 1 mM MgCl_2_, 40 mM KCl, 0.1 mM EDTA, 1 mM DTT and 12% glycerol). To perform the competition assay, excess of unlabeled competitor's oligo was added to the EMSA reaction mixture. Protein–DNA complexes were resolved in 5% polyacrylamide gels containing 0.5 × TBE and exposed to phosphorimager (Bio-Rad Laboratories Inc., Hercules, CA, USA). For the supershift assay, nuclear extracts in EMSA reaction buffer were incubated with different antibodies for 30 min and probes were then added.

### Chromatin immunoprecipitation assay

After treatment with TGF-*β* or overexpression of KLF17, nuclear proteins were cross-linked to genomic DNA by adding formaldehyde for 10 min directly to the medium to a final concentration of 1%. Cross-linking was stopped by adding glycine to a final concentration of 0.125 M and incubating for 5 min at room temperature on a rocking platform. The medium was removed and the cells were washed twice with ice cold phosphate-buffered saline (PBS) (140 mM NaCl, 2.7 mM KCl, 1.5 mM KH_2_PO_4_ and 8.1 mM Na_2_HPO_4_.2H_2_O). The cells were collected by scraping in ice cold PBS supplemented with a protease inhibitor cocktail. After centrifugation, the cell pellets were resuspended in lysis buffer containing 1% SDS, 10 mM EDTA, protease inhibitors and 50 mM Tris–HCl (pH 8.1) and the lysates were sonicated to result in DNA fragments of ~200 to 1000 bp in length. Cellular debris was removed by centrifugation and the lysates were diluted 1 : 10 in ChIP dilution buffer (0.01% SDS, 1.1% Triton X-100, 1.2 mM EDTA, 16.7 mM NaCl, protease inhibitors and 16.7 mM Tris–HCl (pH 8.1)). Non-specific background was removed by incubating the chromatin resuspension with a salmon sperm DNA/protein A agarose slurry for 30 min at 4 °C with agitation. The samples were centrifuged and the recovered chromatin solutions were incubated with 3–5 *μ*g of indicated antibodies overnight at 4 °C with rotation. The immunocomplexes were collected with 60 *μ*l of protein A agarose slurry for 2 h at 4 °C with rotation. The beads were pelleted by centrifugation for 1 min at 4 °C and washed sequentially for 5 min by rotation with 1 ml of the following buffers: low salt wash buffer (0.1% SDS, 1% Triton X-100, 2 mM EDTA, 150 mM NaCl and 20 mM Tris–HCl (pH 8.1)), high salt wash buffer (0.1% SDS, 1% Triton X-100, 2 mM EDTA, 500 mM NaCl and 20 mM Tris–HCl (pH 8.1)) and LiCl wash buffer (0.25 mM LiCl, 1% Nonidet P-40, 1% sodium deoxycholate, 1 mM EDTA and 10 mM Tris–HCl (pH 8.1)). Finally, the beads were washed twice with 1 ml TE buffer (1 mM EDTA and 10 mM Tris–HCl (pH 8.0)). The immunocomplexes were then eluted by adding 250 *μ*l elution buffer (1% SDS and 100 mM NaHCO_3_) and incubation for 15 min at room temperature with rotation. After centrifugation, the supernatant was collected and the cross-linking was reversed by adding NaCl to final concentration of 200 mM and incubating overnight at 65 °C. The remaining proteins were digested by adding proteinase K (final concentration 40 *μ*g/ml) and incubation for 1 h at 45 °C. The DNA was recovered by phenol/chloroform/isoamyl alcohol (25 : 24 : 1) extractions and precipitated with 0.1 volumes of 3 M sodium acetate (pH 5.2) and 2 volumes of ethanol using glycogen as a carrier. PCR amplification of the genomic-fragments was performed with specific primers flanking putative binding sites of target promoters. The PCR products were separated by electrophoresis through 2.0% agarose.

### Production and purification of Smad3 fusion protein

Smad3 protein was purified as described previously.^31^ Full-length Smad3 protein fused to GST were expressed in *Escherichia coli* and partially purified by column chromatography using Pharmacia's protocol. Briefly, bacteria were grown in 2x YTA medium and induced with 0.1 mM IPTG. After sonication, GST fusions were isolated using glutathione–Sepharose 4B, washed three times, eluted and then dialysed against PBS supplemented with 2 mM DTT and 0.5 mM PMSF.

### Luciferase reporter constructs

DNA fragments containing KLF17 and Smad3 genomic sequences were amplified from HepG2 cells genomic DNA using the PCR and ligated into kpn1/xhol sites of the promoterless pGL3-Basic (Promega, Madison, WI, USA) vector.

### Luciferase assay

After transfection and/or treatment, the cells were washed with PBS three times. The cells were then lysed in the luciferase cell culture lysis buffer provided with the Luciferase Assay Kit (Promega). After a brief vortex, whole cell lysates were centrifuged in the cold (4 °C) at 12 000 r.p.m. for 2 min. Supernatant was collected in a fresh tube and 20–30 ml of that was added to luciferase assay substrate (60–80 ml). Luminescence was measured as relative light units, twice for each lysate, taking the reading of luciferase assay using a LUMIstar OPTIMA, BMG LABTECH Inc. (Cary, NC, USA). Each assay was repeated for three times. Fold repression values were represented as mean of the three independent experiments.

### Nuclear extracts preparation

Cells from 100-mm dishes were washed with PBS and scraped. After another washing, cells were suspended in 2 ml of cold buffer A (20 mM HEPES pH 7.9, 20 mM NaF, 1 mM Na_3_VO_4_, 1 mM Na_4_P_2_O_7_, 0.13 *μ*M okadaic acid, 1 mM EDTA, 1 mM EGTA, 0.4 mM ammonium molybdate, 1 mM DTT, 0.5 mM PMSF and 1 *μ*g/ml each leupeptin, aprotinin and pepstatin). Cells were allowed to swell on ice for 15 min and then lysed by 30 strokes of a Dounce all-glass homogenizer (Thomas Scientific, Swedesboro, NJ, USA). Nuclei were pelleted by centrifugation and resuspended in 600 *μ*l of cold buffer C (buffer A, 420 mM NaCl and 20% glycerol). The nuclear membrane was lysed by 15 strokes of a Dounce all-glass homogenizer. The resulting suspension was stirred for 30 min at 4 °C. The clear supernatant was aliquoted and frozen at −80 °C.

### RNA Interference

Cells were cultured to 30% confluence. For each well in a six-well culture dish, 20 nM of Smad3–KLF17 siRNAs, or appropriate negative control siRNAs, were transfected into cells using Lipofectamine 2000 following manufacturer's protocol. Cells were incubated at 37 °C in a CO2 incubator, and 6–8 h later 10% serum growth medium was added to the transfection mixture. Cell extracts were assayed by Western blot for Smad3–KLF17 protein expression at 72 h post transfection, while for mRNA expression at 48 h after transfection. Please see the Supplementary Information for primer sequences.

### Real time-PCR

Total RNA from cells was isolated using TRIZOL (Invitrogen, Life Technologies) following manufacturer's protocol. Briefly, 0.5–1 *μ*g of total RNA was reverse transcribed in a total volume of 25 *μ*l, including 132 U of Moloney-murine-leukemia virus reverse transcriptase, 26.4U of RNAase inhibitor, 0.6 *μ*g of (dT)15 primer, 2 *μ*M dNTPs and 1x Moloney-murine-leukemia virus RT buffer provided by Promega. Aliquots of the RT products were used for RT-PCR analysis. For semi qRT-PCR, 2 *μ*l of RT products was brought to a volume of 25 *μ*l containing 1.5 mM MgCl2, 0.25 mM of each dNTPs, 0.5 *μ*M of both the upstream and downstream PCR primers, and 1x Taq Reaction-Buffer and 1.25 U of Taq DNA polymerase provided by Promega. PCR products were visualized by electrophoresis on a 2% agarose gel in 0.5 × TBE buffer after staining with 0.5 *μ*g/ml ethidium bromide. For qRT-PCR, 2 *μ*l of reverse transcribed cDNA was subjected to RT-PCR using mastermix with SYBR green (Bio-Rad Laboratories Inc.) and the Mx3005P-quantitative RT-PCR system (Stratagene, Amsterdam, Netherlands). Each reaction consisted SYBR green (1 : 60 000 final concentration), 40 nM of both sense and antisense primers, 2 *μ*l of cDNA and H2O to a final volume of 20 *μ*l. Each experiment was performed in duplicates, and repeated thrice.

### Preparation of total cell extract and Western blot analysis

Cells were washed with PBS and treated with an extraction buffer (50 mM Tris–HCl, pH 7.4, 1% Nonidet P-40, 0.25% sodium deoxycholate, 150 mM NaCl and 1 mM EDTA) supplemented with 1 mM phenylmethanesulfonyl fluoride, 1 mM sodium orthovanadate (Na3VO4), 0.1 mM dithiothreitol, 0.4 *μ*g/ml leupeptin/pepstatin. Cell extract was stored at −20 °C until required. Protein samples were subject to electrophoresis in 10% SDS polyacrylamide-gel. Separated proteins were electroblotted to Nitrocellulose membranes (Bio-Rad Laboratories Inc.), and blot was blocked for 1 h at room temperature with blocking buffer 0.1% PBST with 5% fat-free dried milk powder. Blot was then incubated with primary antibodies, (1 : 1000 dilutions) at 4 °C overnight. Blot was washed with 0.1% TBST three times, and incubated with secondary antibodies (mouse, rabbit; 1 : 5000 dilution) for 1 h. Blot was washed again three times and exposed to Odyssey LI-COR-scanner (LI-COR Biotechnology, Lincoln, NE, USA).

### MTT assay

Cell viability was assessed with a MTT assay in replicates. Cells were seeded in 96-well plate at 2.5 × 10^3^ cells/well, and incubated in 10% FBS supplemented with DMEM for 24 h. After that cells were treated with etoposide/TGF-*β* for indicated time points. Controls received DMSO vehicle at a concentration equal to that in drug-treated cells. After that drug containing medium was replaced with 200 *μ*l of 10% FBS supplemented with DMEM containing 0.5 mg/ml MTT, and cells were incubated in the CO_2_ incubator at 37 °C for 2 h, and absorbance (490 nm) was measured and analyzed (Mississauga, ON, Canada).

### Cell cycle analysis

Cell cycle analysis was carried out by estimating DNA contents with flow cytometry. Cells were fixed in ice cold 70% ethanol, incubated overnight at −20 °C and stained with propidium iodide/Triton X-100 containing RNaseA solution (Shanghai Promega Biological Products, Ltd., Shanghai, China) for 15 min at 37 °C. Cell cycle analysis was performed using BD CantoII cell analyzer (Mississauga, ON, Canada).

### Bioinformatics analysis

Microarray data sets were analyzed by arrayQualityMetrics, affyQCReport and affy packages from bioconductor (http://www.bioconductor.org/) with R (http://www.r-project.org/). First, all raw data were downloaded from NCBI Gene Expression Omnibus (GEO, http://www.ncbi.nlm.nih.gov/geo) database (ID:GSE6764). Second, 55 samples were chosen from the data sets and grouped into 4 classes, namely normal samples (*n*=10), cirrhotic patients (*n*=10), early HCC patients (including very early HCC, *n*=18) and advanced HCC patients (including very advanced HCC, *n*=17). The data were normalized by robust multi-array average expression measure depending on affy packages in R. The log2 ratio gene expression values were calculated based on the normalized data.

### Immunohistochemsitry

Paraffin-embedded sections (3*-μ*m thick) of different tumors and adjacent normal tissues were used to perform IHC reaction. Tissue section were de-paraffined with xylene and dehydrated with sequential washes of 100, 95 and 70% ethanol. Endogenous peroxidase activity was quenched using 0.3% hydrogen peroxide in methanol for 30 min and then washed in PBS. Antigen retrieval was achieved using a pressure boiler heating in retrieval solution, pH 6, at 125 °C for 4 min, followed by a 20-min cool down period at room temperature. Slides were then incubated with anti-Smad3 and anti-KLF17 antibodies at 4 °C overnight. Then the slides were rinsed three times in PBS and incubated in biotin-labeled rabbit anti-rabbit secondary antibodies for 1 h at room temperature. After washing three times with PBS, the staining was performed using 3, 3′-diaminobenzidine. Sections were counterstained with hematoxylin. We also compared IHC data between tumors and corresponding adjacent normal tissues by percentage of intensity of staining to estimate the changes between Smad3 and KLF17.

## Abbreviations

ChIP: chromatin immnuoprecipitation
EMSA: electrophoretic mobility shift assay
EMT: epithelial–mesenchymal-transition
HCC: human hepatocellular carcinoma
KLF-17: Kruppel-like-factor-17
KLF17RE: KLF17 responsive elements
MTT: 3-(4,5-dimethylthiazol-2-yl)-2,5-diphenyltetrazolium bromide
PARP: poly (ADP-ribose) polymerase
PBS: phosphate-buffered saline
RNAi: RNA interference
SATB1: sequence-binding protein-1
SBE: Smad binding element
SBE-Luc: SBE-containing luciferase reporter
siRNA: small interference RNA
TGF-*β*1: transforming growth factor-*β*1
