# Supplementary material for: KLF17 empowers TGF-β/Smad signaling by targeting Smad3-dependent pathway to suppress tumor growth and metastasis during cancer progression
Source: Cell Death Dis. 2015 Mar 12;6(3):e1681–. doi: 10.1038/cddis.2015.48 (PMC4385926; doi:10.1038/cddis.2015.48)
Supplement: Supplementary Figures [file cddis201548x1.pdf]

Supplementary Figure 1

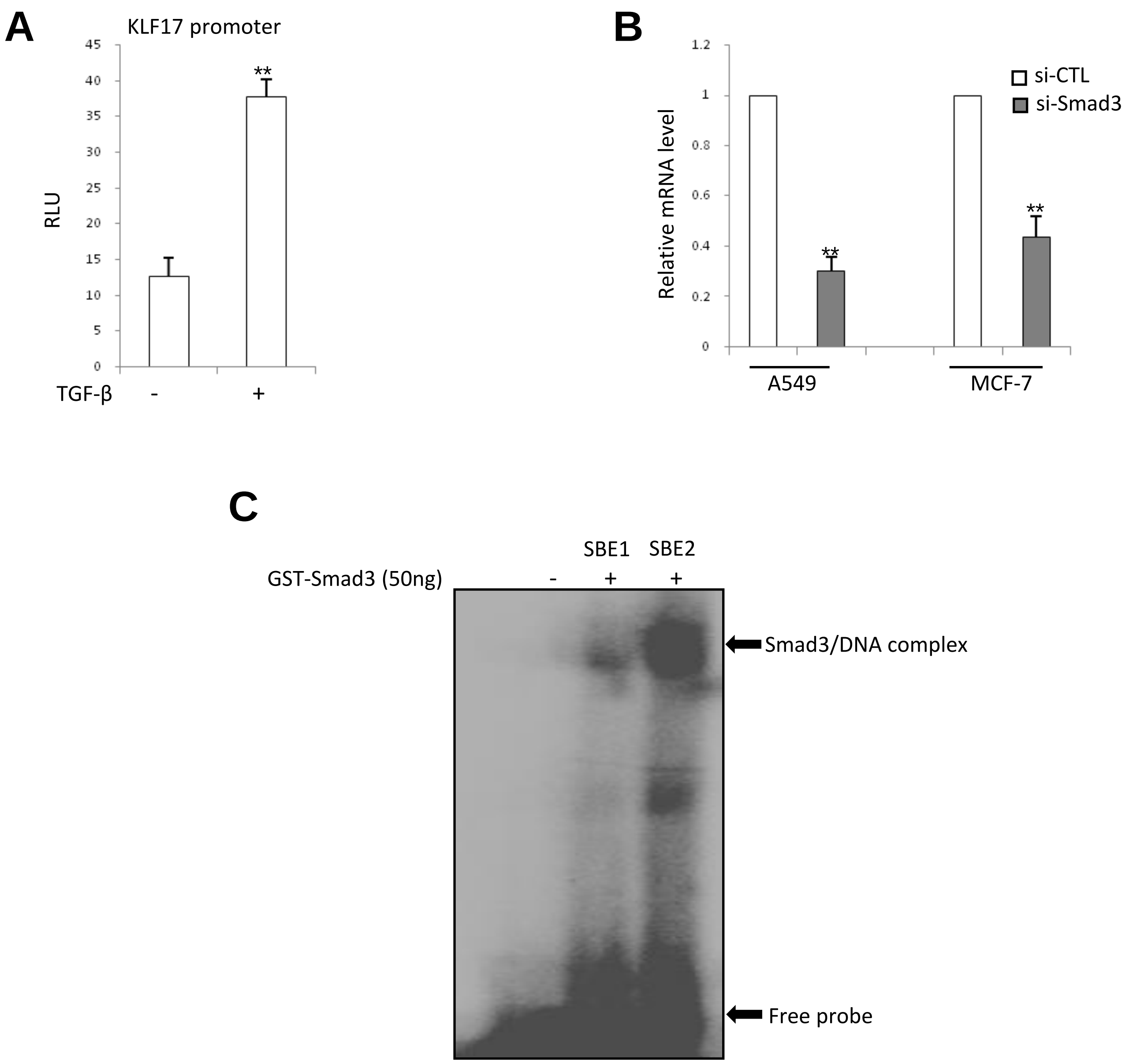

Supplementary Figure 2

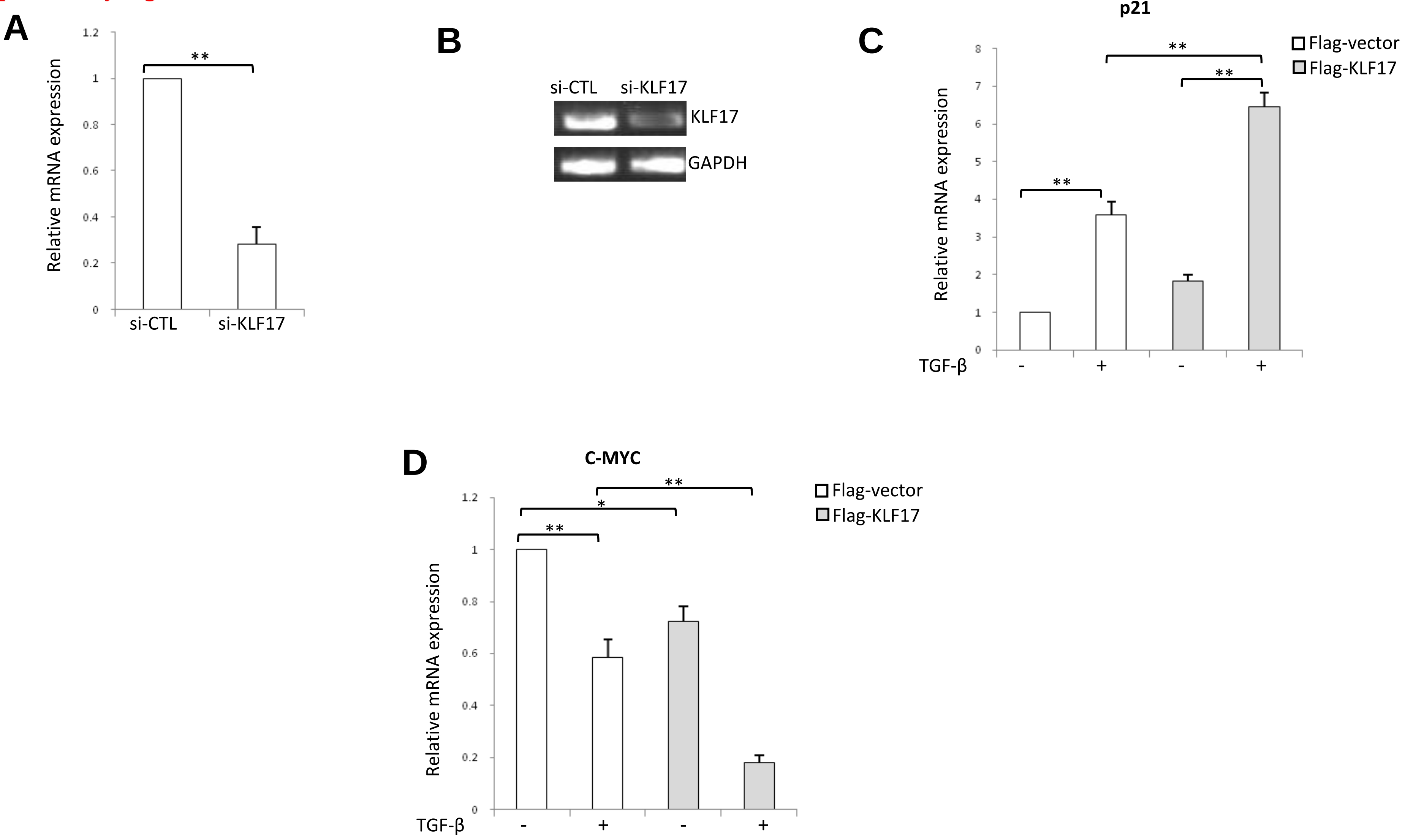

Supplementary Figure 3

A

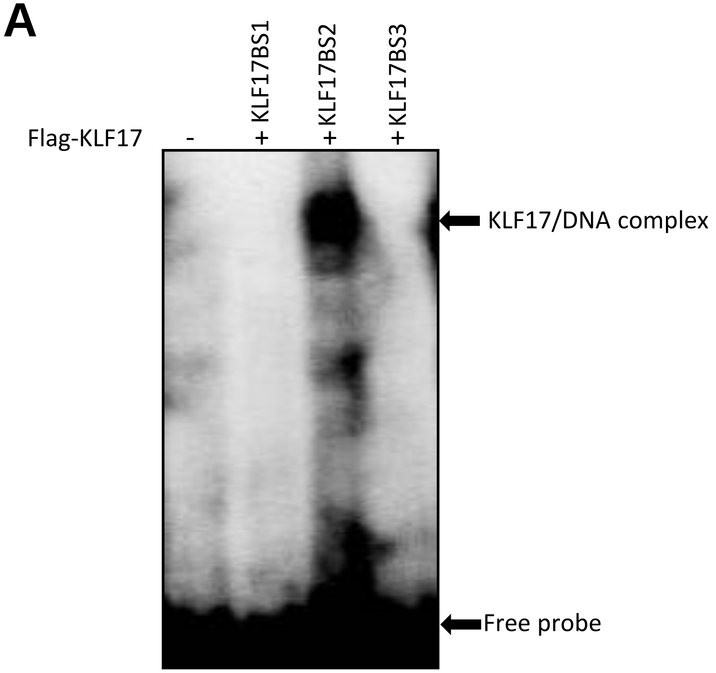

Supplementary Figure 4

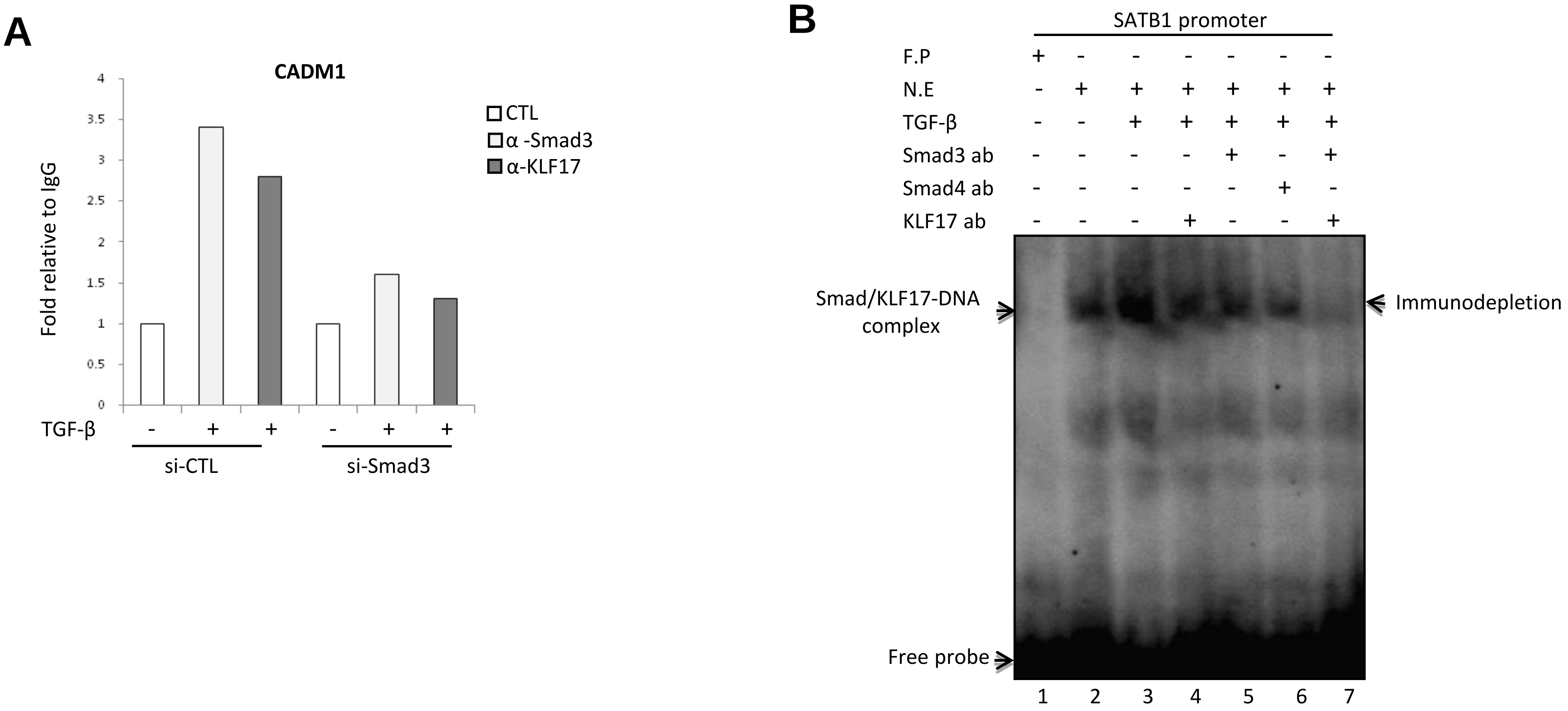

Supplementary Figure 5

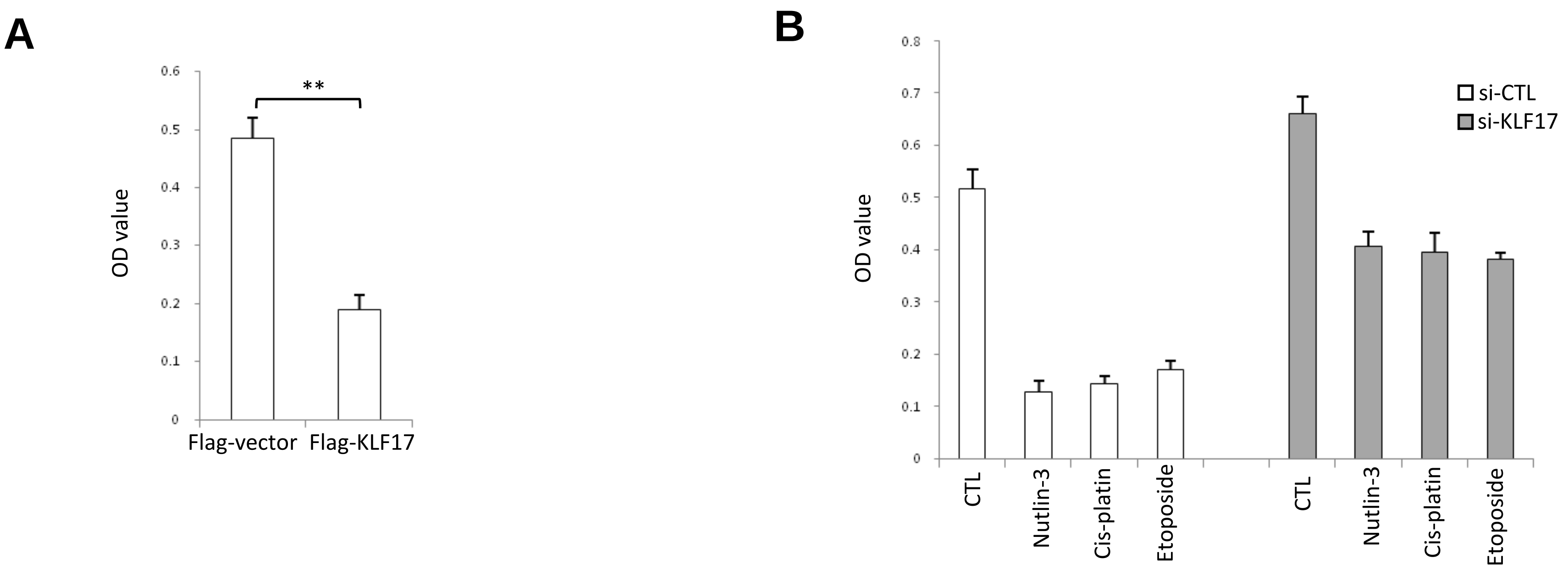

Supplementary Figure 6

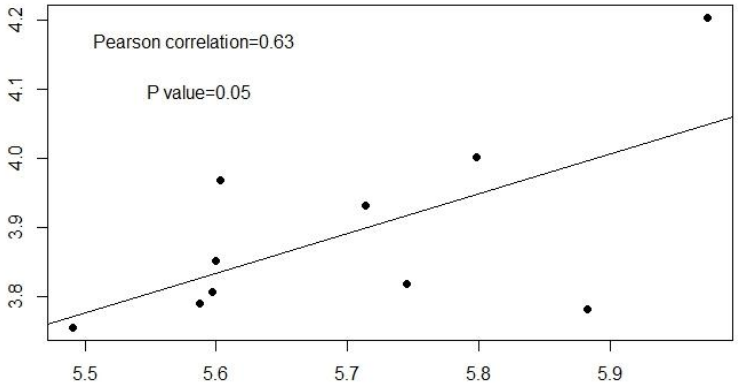

Supplementary Figure 7

Summary of IHC results of KLF17 and SMAD3 expression in multiple human cancer tissue

Cancer type	Sample amount A/C	KLF17 level amount			SMAD3 level amount		
		-	+	++/+++	-	+	++/+++
Liver cancer	A 2	0	0	2	0	0	2
	C 2	1	1	0	2	0	0
Intestinal cancer	A 6	1	1	4	1	2	3
	C 6	4	2		5	1	0
Breast cancer	A 9	1	2	6	2	1	6
	C 7	5	2	0	4	3	0

A: cancer region C: normal region
